# Supplementary material for: Three diverse motives for information sharing
Source: Commun Psychol. 2024 Nov 6;2:107. doi: 10.1038/s44271-024-00144-y (PMC11541573; doi:10.1038/s44271-024-00144-y)
Supplement: Supplementary file 2 — Supplementary Materials [file 44271_2024_144_MOESM2_ESM.pdf]

Supplementary Materials

Supplementary Results

Results are the same when using Linear Mixed-Effect Models. Table S1 shows the results of Linear Mixed-effects Models that predict (a) information-sharing and (b) information-seeking on each trial from three factors: (i) level of uncertainty (equal to 100 minus the algorithms' accuracy), (ii) instrumentality (coded as 1 if information could be used to alter the portfolio and 0 otherwise) and (iii) valence of information (the stocks' value ranged from -400 to -500 and from +400 to +500.

Exp1	Motives	β	95% CI	SE	df	t	p
Information-sharing	Uncertainty	0.49	[0.02, 0.96]	0.24	125.11	2.059	0.041
Information-sharing	Instrumentality	0.77	[0.47, 1.07]	0.15	125.02	5.280	< 0.001
Information-sharing	Valence	0.42	[0.24, 0.6]	0.09	125.11	4.194	< 0.001
Information-seeking	Uncertainty	0.71	[0.35, 1.07]	0.18	121.96	3.93	< 0.001
Information-seeking	Instrumentality	1.46	[1.1, 1.82]	0.18	121.99	8.043	< 0.001
Information-seeking	Valence	0.40	[0.22, 0.58]	0.09	121.63	4.50	< 0.001

Replication Study	Motives	β	95% CI	SE	df	t	p
Information-sharing	Uncertainty	1.03	[0.58, 1.48]	0.23	117.3	4.507	< 0.001
Information-sharing	Instrumentality	0.93	[0.64, 1.22]	0.15	116.96	5.75	< 0.001
Information-sharing	Valence	0.40	[0.18, 0.62]	0.11	116.87	3.65	< 0.001
Information-seeking	Uncertainty	0.96	[0.53, 1.39]	0.23	109.97	4.21	< 0.001
Information-seeking	Instrumentality	1.32	[0.97, 1.67]	0.18	109.99	7.14	< 0.001
Information-seeking	Valence	0.23	[0.09, 0.37]	0.07	109.10	3.28	< 0.001

Table S1. Participants consider the impact of information on affect, action and uncertainty when deciding whether to inform others and when deciding when to seek information for themselves. Beta coefficients predicting informing others and informing self from uncertainty, instrumentality and valence in Exp1 and the Replication experiment are shown. Participants prefer to share and seek information when (i) the receiver's uncertainty was high (ii) when information was instrumental to the receiver, and (iii) when the information would likely convey good news – that is when the expected/true value of the stocks was high.

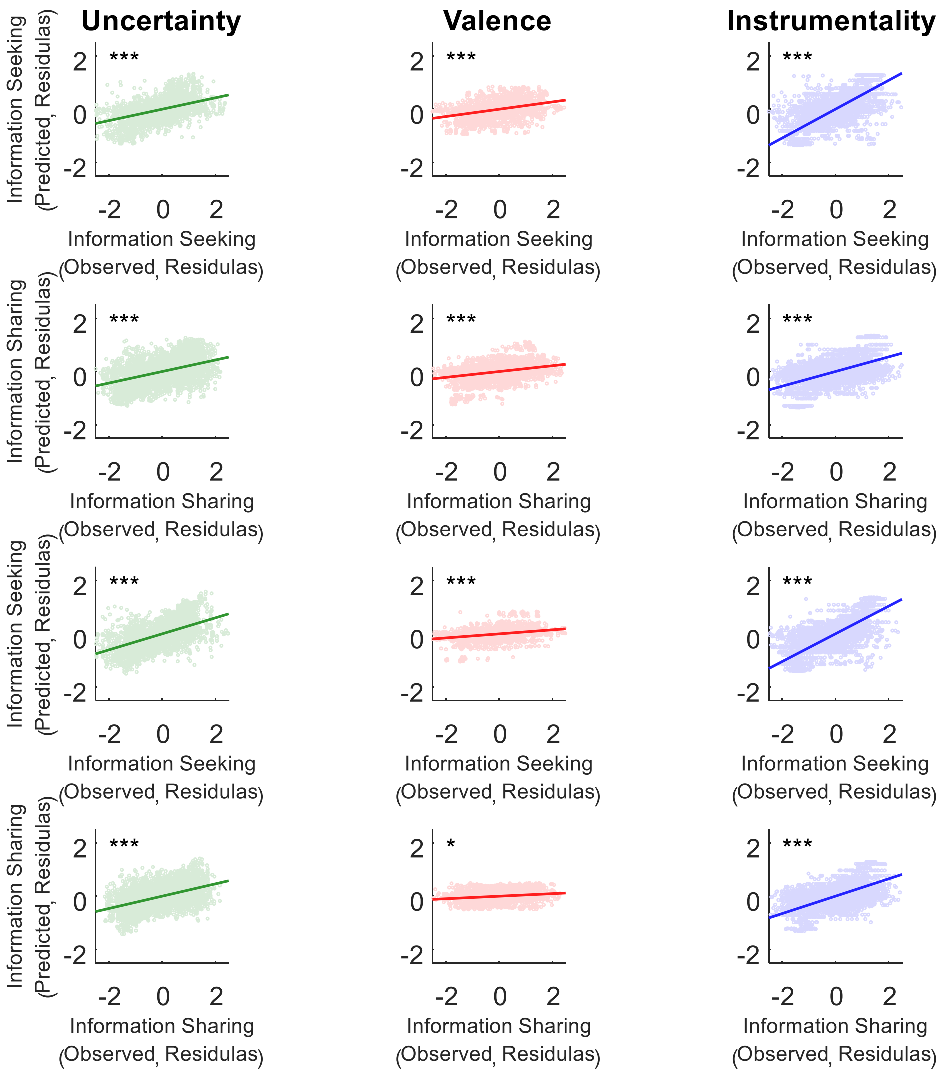

Figure S1. Observed vs. predicted information seeking/sharing. The scatterplots display the relationships between the Observed data (residuals, x-axis) and the predicted information seeking/sharing (residuals, y-axis) using only one motive: Uncertainty (green, left column), Valence (red, middle column), and Instrumentality (blue, right column). The top two rows present data from Exp1 ($N_{Seeking}$ = 114 and $N_{Sharing}$ = 109), while the bottom two rows present data from the Replication ($N_{Seeking}$ = 102 and $N_{Sharing}$ = 100). The predicted information seeking/sharing for each motive was calculated using mixed models, while controlling for the influence of the other two motives (i.e., when predicting information seeking/sharing using one specific motive, the effects of the other two motives were removed). All variables were z-scored. Shaded areas represent the 95% confidence interval. * = p< 0.05; *** = p < 0.001.

We compared the three-factors model which included instrumentality, valence and uncertainty to models including only one or two of the variables. The full model fitted the data better both when informing others and when informing the self, as observed by lower BIC score (Table S2). Note that differences in BIC scores higher than 10 are considered strong evidence in favor of the model with the lower BIC score (Lorah, J., & Womack, A., 2019). The differences between each model and the best (full) model are presented in the column ΔBIC in Table S2.

	Informing Others		Informing Self	
Exp1	BIC	ΔBIC	BIC	ΔBIC
**Intrumentality+Valence+Uncertainty**	**37107.8**		**31468.8**	
Valence	41290.2	4182.4	39422.9	7954.1
Instrumentality	39777.1	2669.3	34354.7	2885.9
Uncertainty	40413.5	3305.7	39042.8	7574
Intrumentality+Uncertainty	37937.6	829.8	32612.8	1144
Valence+Uncertainty	39860.8	2753	38603.8	7135
Intrumentality+Valence	39148.9	2041.1	33461.3	1992.5
Replication				
**Intrumentality+Valence+Uncertainty**	**33449.6**		**28356.9**	
Valence	38476.7	5027.1	35631.8	7274.9
Instrumentality	36507	3057.4	31389.3	3032.4
Uncertainty	37631.8	4182.2	34501	6144.1
Intrumentality+Uncertainty	34511.8	1062.2	28833.4	476.5
Valence+Uncertainty	36983.7	3534.1	34343.1	5986.2
Intrumentality+Valence	35726.5	2276.9	31121	2764.1

Table S2. Information-sharing and information-seeking decisions are best explained by a model that includes the instrumentality of information, its valence and the uncertainty of the receiver, as observed by lower BIC score. For both seeking and sharing information, the model including the three factors fit the data better than models including only one or two factors.

Interactions between the different motives. In the main text, linear regressions were performed for each individual to predict information-sharing and information-seeking on a trial-by-trial basis using uncertainty, instrumentality, and valence as predictors. To further investigate potential interactions between these factors, we extended the model to include two-way and three-way interactions. As shown in **Table S3**, all main factors remained significant. The only interaction that consistently reached statistical significance across Exp. 1 and the replication was the interaction between instrumentality and valence in the sharing condition. The impact of valence was greater when there was no instrumentality as compared to when there was instrumentality to information. Specifically, when instrumentality equals 0, the beta coefficient of valence is significantly higher (M=0.13 compared to M=0.06, t(113) = 2.15, p = 0.03). The same result is obtained for the replication: (0.09 compared to 0.03, t(101) = 2.00, p = 0.047).

	**Exp. 1 – Seek**	**Exp. 1 – Share**	**Replication – Seek**	**Replication – Share**
**Uncertainty****	$p < 0.001$<br>$M_{beta} = 0.13$<br>$BF_{10} = 134.71$<br>$BF_{01} = 0.007$	$p = 0.02$<br>$M_{beta} = 0.09$<br>$BF_{10} = 1.53$<br>$BF_{01} = 0.65$	$p < 0.001$<br>$M_{beta} = 0.19$<br>$BF_{10} = 936.49$<br>$BF_{01} = 0.001$	$p < 0.001$<br>$M_{beta} = 0.18$<br>$BF_{10} = 2930.11$<br>$BF_{01} = 3.41 \times 10^{-4}$
**Valence****	$p < 0.001$<br>$M_{beta} = 0.11$<br>$BF_{10} = 7522.67$<br>$BF_{01} = 1.32 \times 10^{-4}$	$p < 0.001$<br>$M_{beta} = 0.10$<br>$BF_{10} = 969.32$<br>$BF_{01} = 0.001$	$p = 0.002$<br>$M_{beta} = 0.06$<br>$BF_{10} = 14.50$<br>$BF_{01} = 0.69$	$p < 0.001$<br>$M_{beta} = 0.06$<br>$BF_{10} = 68.26$<br>$BF_{01} = 0.01$
**Instrumentality****	$p < 0.001$<br>$M_{beta} = 0.39$<br>$BF_{10} = 1.40 \times 10^{14}$<br>$BF_{01} = 7.13 \times 10^{-15}$	$p < 0.001$<br>$M_{beta} = 0.21$<br>$BF_{10} = 4.21 \times 10^{5}$<br>$BF_{01} = 2.37 \times 10^{-6}$	$p < 0.001$<br>$M_{beta} = 0.33$<br>$BF_{10} = 3.37 \times 10^{10}$<br>$BF_{01} = 2.96 \times 10^{-11}$	$p < 0.001$<br>$M_{beta} = 0.26$<br>$BF_{10} = 1.19 \times 10^{7}$<br>$BF_{01} = 8.37 \times 10^{-8}$
**Uncertainty X Valence**	$p < 0.001$<br>$M_{beta} = -0.07$<br>$BF_{10} = 62.68$<br>$BF_{01} = 0.016$	$p = 0.11$<br>$M_{beta} = 0.01$<br>$BF_{10} = 0.36$<br>$BF_{01} = 2.76$	$p = 0.08$<br>$M_{beta} = -0.03$<br>$BF_{10} = 0.50$<br>$BF_{01} = 1.97$	$p = 0.22$<br>$M_{beta} = -0.01$<br>$BF_{10} = 0.22$<br>$BF_{01} = 4.44$
**Uncertainty X Instrumentality**	$p = 0.06$<br>$M_{beta} = -0.03$<br>$BF_{10} = 0.60$<br>$BF_{01} = 1.66$	$p = 0.67$<br>$M_{beta} = 0.006$<br>$BF_{10} = 0.11$<br>$BF_{01} = 8.81$	$p = 0.09$<br>$M_{beta} = -0.03$<br>$BF_{10} = 0.45$<br>$BF_{01} = 2.19$	$p = 0.35$<br>$M_{beta} = 0.01$<br>$BF_{10} = 0.16$<br>$BF_{01} = 5.94$
**Instrumentality X Valence***	$p = 0.28$<br>$M_{beta} = -0.01$<br>$BF_{10} = 0.18$<br>$BF_{01} = 5.42$	$p = 0.03$<br>$M_{beta} = -0.03$<br>$BF_{10} = 0.97$<br>$BF_{01} = 1.03$	$p = 0.78$<br>$M_{beta} = 0.003$<br>$BF_{10} = 0.11$<br>$BF_{01} = 8.71$	$p = 0.04$<br>$M_{beta} = -0.03$<br>$BF_{10} = 0.81$<br>$BF_{01} = 1.22$
**Uncertainty X Valence X Instrumentality**	$p = 0.06$<br>$M_{beta} = 0.02$<br>$BF_{10} = 0.57$<br>$BF_{01} = 1.74$	$p = 0.03$<br>$M_{beta} = -0.02$<br>$BF_{10} = 1.01$<br>$BF_{01} = 0.98$	$p = 0.11$<br>$M_{beta} = 0.02$<br>$BF_{10} = 0.37$<br>$BF_{01} = 2.67$	$p = 0.03$<br>$M_{beta} = 0.02$<br>$BF_{10} = 1.05$<br>$BF_{01} = 0.95$

Table S3. Models with interactions. The mean beta values of uncertainty, valence, instrumentality and their interactions in the information seeking/sharing in Exp1 and the replication. For each beta vales, the p-value from its comparison against 0 is provided, along with the Bayes factors: BF_{10} (the level of support in favor of the alternative hypothesis) and BF_{01} (the level of support in favor of the null hypothesis). ** = a *replicable* effect which is observed across seeking and sharing. *= a *replicable* effect across sharing.

It is important to note that the analysis of the interactions between the motives was exploratory and deviated from the pre-registration report. As a result, the interaction results should be interpreted with caution and further validated in future studies. Moreover, most of the interactions were on the verge of significance, raising the question of whether the non-significant results were due to a lack of statistical power or the genuine absence of an effect. To address this issue, we conducted a Bayesian analysis comparing the evidence for the null hypothesis (H0) to that of the alternative hypothesis (H1).

A Bayes factor (BF_{10}) greater than 1 indicates support for the alternative hypothesis, while a Bayes factor less than 1 favors the null hypothesis. The magnitude of the Bayes factor measures the strength of evidence, with larger values indicating stronger support for one hypothesis over the other. Specifically, a BF_{10} between 1 and 3 indicates weak evidence, a BF_{10} between 3 and 10 indicates moderate evidence, and a BF_{10} greater than 10 indicates strong evidence in favor of the alternative hypothesis (van Doorn et al., 2021). Conversely, a BF_{01} (the reciprocal of BF_{10}) between 1 and 3 indicates weak evidence, a BF_{01} between 3 and 10 indicates moderate evidence, and a BF_{01} greater than 10 indicates strong evidence in favor of the null hypothesis.

Table S3 presents the Bayes factors for the interactions between uncertainty, valence, and instrumentality. The null hypothesis was supported for the interactions between Uncertainty X Valence (Sharing/Replication), Uncertainty X Instrumentality (Sharing), and Instrumentality X Valence (Seeking). However, for all other interactions, the Bayes factors yielded inconclusive results, suggesting that higher-powered designs are needed to draw valid conclusions about these effects.

Participants explicitly stated that they shared information to help other players. At the end of Exp1 67 participants were asked ("How did you decide whether to open the envelope for the other participants?"). In response most participants explicitly referenced the other participant (using words such as 'they', 'them', 'others' or 'the participant') and many explicitly considered the instrumental and non-instrumental impact of the shared information on the other participant (using words like 'surprise', 'disappointment', 'good news', 'bad news', value of 'knowing'). Most expressed a desire to help the other.
Below a few representative responses:

- "In the first task my decision was based off whether *the participants would be disappointed or pleased*. The second task was whether *they should risk investing their money*."
- "Good news vs bad news, reinforce if *they were doing well* and encourage where performing above expectations."
- "I wanted to give the participants the most info I could."
- "I'm a nice person if it made a difference, I wanted them to see it, the first part it didn't matter so I didn't care."
- "Mostly if the participant would have the chance to move from a negative scenario to a positive which was possible *only with my help*."
- "To *help* others out without making them feel bad"

These responses suggest that participants aimed to help others by providing information they believed would be beneficial to them (in terms of money, emotion, etc.). This is consistent with vast research demonstrating that people often help others even if there is no material benefit to them (Krebs, 1975; Warneken, F., & Tomasello, M. 2009)."

We quantified the above interpretation by having two naïve observers independently rate all responses to indicat whether the responder:

(1) believed they were interacting with another participant. (Options: (a) the responder believes they are interacting with another participant; (b) the responder does not believe they are interacting with another participant; (b) no indication either way).

(2) is trying to help the other participant. (Options: (a) the responder is trying to help the other participant; (b) the responder is not trying to help the other participant; (c) no indication either way).

The observers showed strong agreement between them (average absolute ICCs was 0.97 for question 1 and 0.96 for question 2). Analysis of the ratings of the first question revealed that 56.5% of responders explicitly indicated that they believed they were interacting with another participant; 0% of responders explicitly indicated that they did not believe they were interacting with another participant; 43.5% of responses were not informative either way. Analysis of the ratings of the second question revealed 51.5% of responses explicitly demonstrated a desire to help; 2.9% of responders explicitly indicated no desire to help; 45.6% of responses were not informative either way.

We compared the beta values of participants who explicitly referenced another participant in their debrief and those who did not. There was no difference in the weight assigned to valence ($t(65) = 1.29$, $p = 0.2$) nor to uncertainty ($t(65) = -0.07$, $p = 0.94$). Participants who expressed explicit belief in the manipulation assigned greater weight to instrumentality upon sharing information ($Mean\ \beta_{Instrumentality} = 0.28$, $SE = 0.06$) than those who did not express explicit belief ($Mean\ \beta_{Instrumentality} = 0.03$, $SE = 0.03$, difference between the two groups: $t(65) = -2.39$, $p = 0.02$) . Similar results were obtained when comparing betas of participants who explicitly expressed their goal as helping others than those that did not. No differences were found for valence betas ($t(65) = 1.53$, $p = 0.13$) or uncertainty betas ($t(65) = 0.18$, $p = 0.86$). Participants who explicitly expressed their goal as helping others assigned greater weight to instrumentality upon sharing information ($Mean\ \beta_{Instrumentality} = 0.28$, $SE = 0.06$) than those who did not ($Mean\ \beta_{Instrumentality} = 0.04$, $SE = 0.03$; $t(65) = -3.25$, $p = 0.002$) .

Supplementary Methods

Exp1	Mean R	95% CI	t	df	p
**Information-sharing**					
Uncertainty and Valence	0.009	[-0.01, 0.03]	0.900	124	0.370
Uncertainty and Instrumentality	-0.007	[-0.03, 0.01]	-0.679	124	0.498
Valence and Instrumentality	0.000	[-0.02, 0.02]	-0.007	124	0.995
**Information-seeking**					
Uncertainty and Valence	0.001	[-0.02, 0.02]	0.114	121	0.909
Uncertainty and Instrumentality	0.007	[-0.01, 0.03]	0.648	121	0.518
Valence and Instrumentality	-0.008	[-0.03, 0.01]	-0.888	121	0.376
**Replication**					
**Information-sharing**					
Uncertainty and Valence	-0.009	[-0.03, 0.01]	-0.862	116	0.391
Uncertainty and Instrumentality	-0.014	[-0.04, 0.01]	-1.437	116	0.153
Valence and Instrumentality	0.007	[-0.02, 0.03]	0.697	116	0.487
**Information-seeking**					
Uncertainty and Valence	-0.009	[-0.03, 0.01]	-0.869	109	0.387
Uncertainty and Instrumentality	-0.002	[-0.02, 0.02]	-0.173	109	0.863
Valence and Instrumentality	-0.006	[-0.02, 0.01]	-0.599	109	0.551

Table S4. Valence, instrumentality and uncertainty were not correlated across trials. For each individual we correlated valence, instrumentality and uncertainty level of information across trials. Correlation coefficients were compared to zero using a t-tests – none were significant.

Supplementary References

1. Lorah, J., & Womack, A. (2019). Value of sample size for computation of the Bayesian information criterion (BIC) in multilevel modeling. *Behavior research methods, 51*, 440-450.
